# Polycomb response elements reduce leaky expression of Cas9 under temperature-inducible *Hsp70Bb* promoter in *Drosophila melanogaster*

**DOI:** 10.1093/g3journal/jkad024

**Published:** 2023-01-27

**Authors:** Natalie Warsinger-Pepe, Carly Chang, Connor R Desroberts, Omar S Akbari

**Affiliations:** School of Biological Sciences, Department of Cell and Developmental Biology, University of California, San Diego, La Jolla, CA 92093, USA; School of Biological Sciences, University of California, San Diego, La Jolla, CA 92093, USA; School of Biological Sciences, University of California, San Diego, La Jolla, CA 92093, USA; School of Biological Sciences, Department of Cell and Developmental Biology, University of California, San Diego, La Jolla, CA 92093, USA

**Keywords:** inducible, Cas9, heat-shock, PREs, polycomb response elements, *Drosophila melanogaster*

## Abstract

Heat-shock-inducible expression of genes through the use of heat-inducible promoters is commonly used in research despite leaky expression of downstream genes of interest without targeted induction (i.e. heat shock). The development of non-leaky inducible expression systems is of broad interest for both basic and applied studies, to precisely control gene expression. Here we characterize the use of Polycomb response elements and the inducible *Heat-shock protein 70Bb* promoter, previously described as a non-leaky inducible system, to regulate Cas9 endonuclease levels and function in *Drosophila melanogaster* after varying both heat-shock durations and rearing temperatures. We show that Polycomb response elements can significantly reduce expression of *Cas9* under *Heat-shock protein 70Bb* promoter control using a range of conditions, corroborating previously published results. We further demonstrate that this low transcript level of heat-induced *Cas9* is sufficient to induce mutant mosaic phenotypes. Incomplete suppression of an inducible *Cas9* system by Polycomb response elements with no heat-shock suggests that further regulatory elements are required to precisely control *Cas9* expression and abundance.

## Introduction

Temporal control of expression through inducible gene expression systems (IGESs) is a useful genetic strategy to understand gene function. One commonly utilized IGES is the heat-shock response system, whereby a heat-shock promoter is placed upstream of a transgene, or gene of interest, which can then be activated by endogenous heat-shock proteins when the environment temperature shifts from lower temperatures to 37°C (heat shock) (Pelham 1982; Lindquist 1984). Despite the power and utilization of this tool, the heat-shock promoter exhibits leaky expression, where promoter activity is seen at temperatures below 37°C (Lindquist 1984; Curtin *et al.* 2008; Naidoo and Young 2012; Fujita *et al.* 2014; Sloan and Barres 2014; Akmammedov *et al.* 2017; Nandy *et al.* 2019). The *Drosophila Heat-shock protein 70Bb* (*Hsp70Bb*) promoter is regularly used for temporal control of gene expression through heat shock in many organisms despite its leakiness (Corces *et al.* 1981; Pelham 1982; Pelham and Bienz 1982; Spena *et al.* 1985; Wei *et al.* 1986; Kust *et al.* 2014). Akmammedov and colleagues utilized *bithoraxoid* (*bxd*) Polycomb response elements (PREs) (Simon *et al.* 1993), which silence adjacent genes through Polycomb group (PcG) protein deposition of H3K27me3 silencing marks (Beisel and Paro 2011), upstream of the *Hsp70Bb* promoter to significantly suppress leaky expression of a downstream gene, *lacZ* (Akmammedov *et al.* 2017). Despite this significant reduction in leaky expression, PRE repeats appear to not be widely used.

Suppressing leaky expression of genes under *Hsp70Bb* promoter control has the potential to aid in basic biological studies of pathway mechanisms as well as applied systems for pest control. Temporal and spatial control of gene expression can aid in understanding details of gene and/or pathway function by regulating timing and/or dosage of gene transcription and products. Temporal control of sterile insect technologies like precision-guided sterile insect technique (pgSIT) can be a powerful and financially impactful tool for scaling up large populations of pest species for release and population control (Kandul *et al.* 2019, 2021, 2022). Heat-shock-inducible *Cas9* has been used in multiple organisms to induce programed mutations, including in rice (Nandy *et al.* 2019) and *Drosophila* (Kandul *et al.* 2021); however these systems still reveal *Cas9* activity without heat shock. In this study, we characterize the effectiveness of PREs upstream *Hsp70BbCas9* in suppressing leaky *Cas9* expression without heat shock. We characterize the ability of *Hsp70BbCas9* to induce mutations in the presence of guide RNAs (gRNAs) against three separate gene targets (*w*, *ey*, *Ser*) after varying heat-shock durations (0, 30 min, 1 h, 2 h), and at three different rearing temperatures (18°C, 21°C, 26°C). Through this genetic characterization, we show that PREs are able to drastically reduce *Cas9* expression under the majority of conditions tested, yet low levels of functional Cas9 are often sufficient to induce mutant mosaic phenotypes.

## Materials and methods

### Fly husbandry and strains

*D. melanogaster* stocks were either reared in 18°C, 21°C, or 26°C incubators with standard light/dark cycles on Texas media from the UCSD *Drosophila* Recharge facility (Fly Kitchen). Stocks were reared at their subsequent temperature for at least three generations before being utilized in experiments. The following fly stocks from the Bloomington *Drosophila* Stock Center were used: p{TKO.GS02468}attP40/II “*sgRNA: w*” (BDSC 79543), p{WKO.1-G12}attP40/II “*sgRNA: ey*” (BDSC 82495), M{WKO.p1-B12}ZH-86Fb/TM3, Sb^1^ “*sgRNA: Ser*” (BDSC 84169), *w^1118^*; PBac{y ^+ mDint2^ = vas-Cas9}VK00027 “*vasaCas9*” (BDSC 51324), y[1] M{w ^+ mC^ = nos-Cas9.P}ZH-2A w* “*nosCas9*” (BDSC 54591). P{y[ + t7.7] = CaryP}attP2. *Hsp70BbCas9* was generated by Nikolay Kandul and Junru Liu (BDSC 92793). PRE-Hsp70Bb-Cas9 plasmid was generated as described below. Embryo injections to generate both *Hsp70BbCas9* and *PRE-Hsp70BbCas9* transgenic animals were carried out at Rainbow Transgenic Flies, Inc. (http://www.rainbowgene.com) using ɸC31-mediated integration into the same genomic attP2 location (BDSC # 8622) and balanced using standard balancer chromosomes. All stocks are listed in the Supplementary Reagents Table.

### Transgene construction

For construction of PRE-Hsp70Bb-Cas9, Hsp70Bb-Cas9-T2A-eGFP (Kandul *et al.* 2021) was PCR amplified with NEB Q5 High-Fidelity 2X Master Mix (M0492) using primers 1157_onestep_p1 and 1157_onestep_p2. PRExpress (Akmammedov *et al.* 2017) was obtained from Addgene (122486). The PRE repeats of PRExpress were removed using Kpn-I and Nhe-I and purified using Zymoclean Gel DNA Recovery Kit (Genesee Scientific #11-301). This fragment was subcloned with the above PCR fragment using Gibson enzymatic assembly (Gibson *et al.* 2009) to generate PRE-Hsp70BbCas9_1.0 (Addgene 190795). Gypsy insulator elements were subsequently cloned into PRE-Hsp70BbCas9_1.0 through two Gibson cloning events to generate PRE-Hsp70BbCas9_1.2 (Addgene 190796) with one gypsy insulator element (KpnI digest, PCR with 1157A_onestep_for and 1157A_onestep_rev) and subsequently PRE-Hsp70BbCas9_1.3 (Addgene 190797) with two gypsy insulator elements (AfeI digest, PCR with 1157B_onestep_for and 1157B_onestep_rev). This final product, PRE-Hsp70BbCas9_1.3 (referred to as PRE-Hsp70Bb-Cas9), was used for transgenesis. Plasmid sequences were verified through Sanger sequencing to ensure the only differences between Hsp70Bb-Cas9-T2A-eGFP and PRE-Hsp70BbCas9_1.3 were the presence of the PREs and gypsy insulator elements (Supplementary Fig. 1). All Sanger sequencing was performed by either Retrogen, Inc. or GENEWIZ, Inc. All primers and plasmids are listed in the Supplementary Reagents Table. At every cloning step, the PRE repeat size was verified (∼2.5 kb) through restriction digestion with Kpn-I and Xho-I, as these repeats are often unstable.

### Mutant generation with cell-specific promoters

To observe mutant phenotypes from the chosen gRNA strains, gRNA stocks were crossed to either *nanos-Cas9* (*nosCas9*) or *vasaCas9* stocks, and the rate of mutant phenotype generation was assessed. All crosses were performed at 26°C. Five virgin female P0 were crossed to five male P0 per vial (for 1–2 vals) and flipped to new vials after one week. All emerged F1 were scored from both vials ∼15 days after initial pairing. F1 were scored for sex, genotype, and visible mutant phenotypes.

### Heat-shock-induced mutant generation

To generate Cas9-mediated mutant *D. melanogaster*, the Cas9 strains were crossed with a gRNA strain to produce either double heterozygous F1 (*gRNA/+*; *Hsp70BbCas9/+*) or trans-heterozygous F1 (*Hsp70BbCas9/gRNA*) (both referred to as trans-heterozygous F1). Five virgin female P0 were crossed to five male P0 per vial and allowed to mate and lay eggs. Crosses at 18°C were allowed to seed a vial for four days, crosses at 21°C were allowed to seed a vial for three days, and crosses at 26°C were allowed to seed a vial for 2 days before P0 were flipped to new vials. All crosses performed at a specific temperature (18°C, 21°C, 26°C) utilized Cas9 stocks reared at the same temperature, unless otherwise specified. Vials with F1 embryos/first instar larvae were submerged in a 37°C water bath for heat-shock durations of either 30 min, 1 h, or 2 h, then returned to their original rearing temperatures. All F1 were examined and scored for sex, genotype, and visible mutant phenotypes using a Leica M165FC fluorescent stereo microscope either after ∼27 days from initial pairing for 18°C rearing, ∼20 days for 21°C rearing, or ∼15 days for 26°C rearing.

### Sample collection and reverse transcription quantitative PCR

All primers used for reverse transcription quantitative PCR (RT-qPCR) are listed in the Supplementary Reagents Table. Adult virgin female flies were used for RT-qPCR to maximize RNA extraction. Virgin females were collected and aged for 6–7 days. Seven to 10 females per condition were pooled together, heat shocked at 37°C as described above, and allowed to recover for 30 min at their original rearing temperature (i.e. 18°C, 21°C, 26°C). The flies were anesthetized, transferred to Eppendorf tubes on ice and immediately homogenized in QIAzol (Qiagen 79306) and stored at −20°C. RNA was extracted from samples following standard TRIzol/chloroform RNA extraction protocol (see Thermo Fisher Scientific TRIzol User Guide). RNA was quantified using a NanoDrop 2000 (Thermo Fisher Scientific) and diluted to 250 ng/μl, aliquoted, and stored at −80°C. About 1 μg of RNA was then treated with DNaseI (ThermoFisher Scientific #89836) to remove any DNA contamination, following a standard protocol. cDNA was synthesized using 5 μl of DNaseI-treated RNA using RevertAid First Strand cDNA Synthesis Kit (ThermoFisher Scientific #K1622) and Oligo (dT)_18_ primers. qPCR was performed using 2x qPCRBIO SyGreen Blue Mix Separate-ROX (PCR Biosystems #PB20.17) on a LightCycler® 96 Instrument (Roche). cDNA samples from 21°C rearing with 2 h heat-shock samples were serially diluted to generate standard curves for each amplified gene fragment and to test primer performance (Supplementary Fig. 2a and b). Undiluted samples (d0) were used for relative quantification to account for possible low Cas9 expression levels for some samples. RT-qPCR reactions (20 μl) were run following 2x qPCRBIO SyGreen Blue Mix Separate-ROX (PCR Biosystems #PB20.17) protocol with 2 μl of sample. Negative controls were run with 2 μl of nuclease-free water. At least three technical replicates were run per plate for each of three biological replicates. Data from 2–3 technical replicates for all three biological replicates were analyzed in LightCycler® 96 software (Roche), Google Sheets, and GraphPad Prism 9. Relative ratios of *Cas9* RNA levels normalized to *RpL32* were calculated using E*_RpL32_*^RpL32_Ct^/E*_Cas9_*^Cas9_Ct^. Normalized expression ratio (or fold change) was also calculated using the ΔΔCt method.

### Genotyping target loci

Control flies (*w^1118^* and heterozygous flies) and trans-heterozygous flies with or without visible mutant phenotypes (or lethal pupae) were separately collected in Eppendorf tubes and frozen at −20°C. Genomic DNA was extracted from individual flies following a standard protocol. In short, single flies were homogenized in 30 μl 10 mM Tris-Cl buffer pH 8.0 with 1 mM EDTA, 25 mM NaCl, and 200 μg/ml Proteinase-K (proK) Qiagen solution (from Qiagen #69504). Samples were incubated at 37°C for 30 min and then at 95°C for 4 min. Samples were either frozen immediately and/or used for PCR amplification. PCR amplification of all target regions was performed using LongAmp Taq 2X Mastermix (NEB #M0287). Amplicon size was verified using gel electrophoresis, and amplicons were purified using QIAquick PCR Purification Kit (Qiagen # 28104) before Sanger sequencing through Retrogen Inc. Primers used can be found in the Supplementary Reagents Table. All sequence file chromatograms were verified and aligned to the following NCBI Reference Sequences: NC_004353.4 (*ey*), NT_033777.3 (*Ser*) (Larkin *et al.* 2021) using SnapGene® 4. Sanger sequencing of target loci was used to identify solely the presence and not type of mutations at or near the gRNA target site(s).

### Microscopy

Representative images of mutant phenotypes and lethal pupae were obtained using light microscopy on a Leica M165FC fluorescent stereomicroscope equipped with the Leica DMC2900 camera. Scale bars were designed manually with a ruler in each original image. Images were then processed for ease of viewing and assembled with Adobe Photoshop.

### Statistical analysis

Percentage of mutant formation for heat-shock-induced mutant generation (Figs. 3, 4 and 5) was calculated using the total number of flies scored for a genotype/condition over the total number of flies scored (combining multiple vials) to minimize any effect by variability across vials or crosses. This percentage was reported along with the standard error of the mean. Significance was calculated using unpaired two-tailed t-test with Welch’s correction using GraphPad Prism9. Significance for all RT-qPCR data was calculated using unpaired two-tailed t-test with Welch’s correction using GraphPad Prism9.

**Fig. 1.**
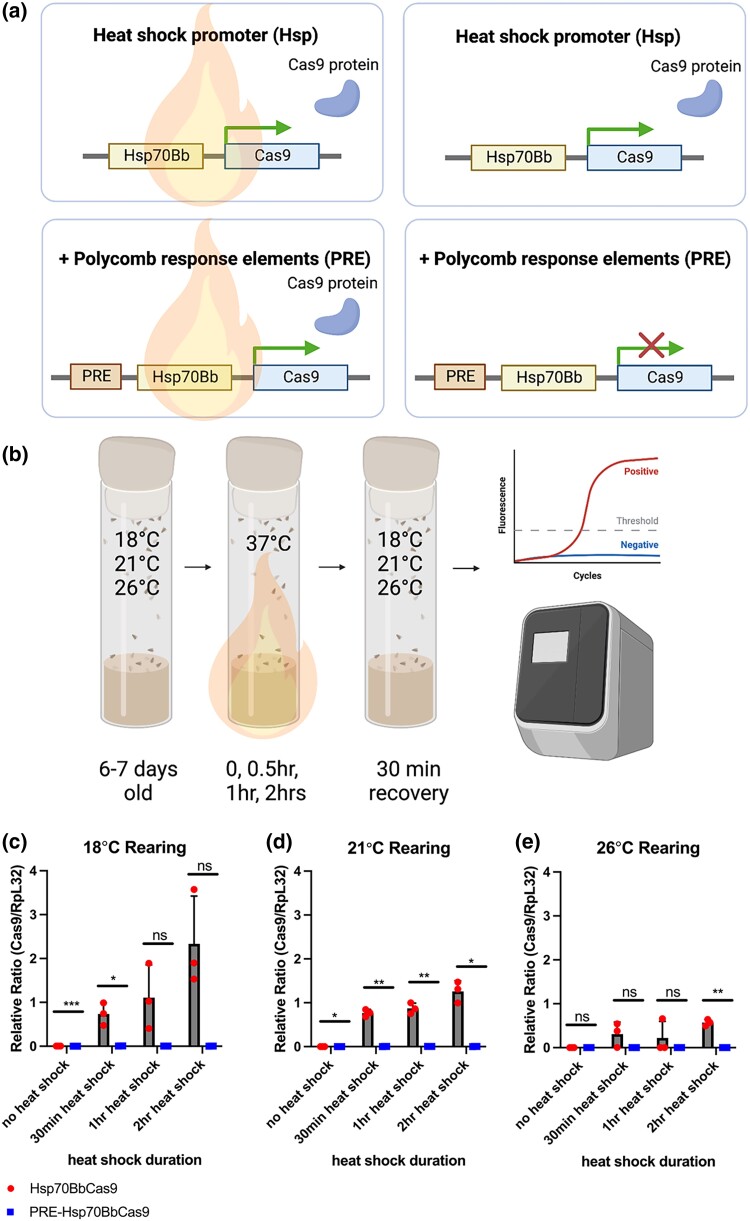
Fold change of *Cas9* RNA levels by RT-qPCR comparing the lack of upstream PRE to having PREs, considering varying heat-shock durations and rearing temperatures. a) Conceptual use of PREs to suppress leaky expression of *Cas9* under heat-shock-inducible *Drosophila melanogaster Hsp70Bb* promoter. Created with BioRender.com. b) Methodology for sample collection and RT-qPCR. Created with BioRender.com. Relative ratio (E*_RpL32_*^RpL32_Ct^/E*_Cas9_*^Cas9_Ct^) of *Cas9*/*RpL32* transcripts at increasing heat-shock durations comparing *Hsp70BbCas9* to *PRE-Hsp70BbCas9* for c) 18°C rearing temperature, d) 21°C rearing temperature, and e) 26°C rearing temperature. Red = *Hsp70BbCas9*, blue = *PRE-Hsp70BbCas9*. Error bars = standard deviation. Significance calculated using unpaired two-tailed t-test with Welch’s correction. ns = *P* > 0.05, * = *P* ≤ 0.05 ** = *P* ≤ 0.01, *** = *P* ≤ 0.001.

**Fig. 2.**
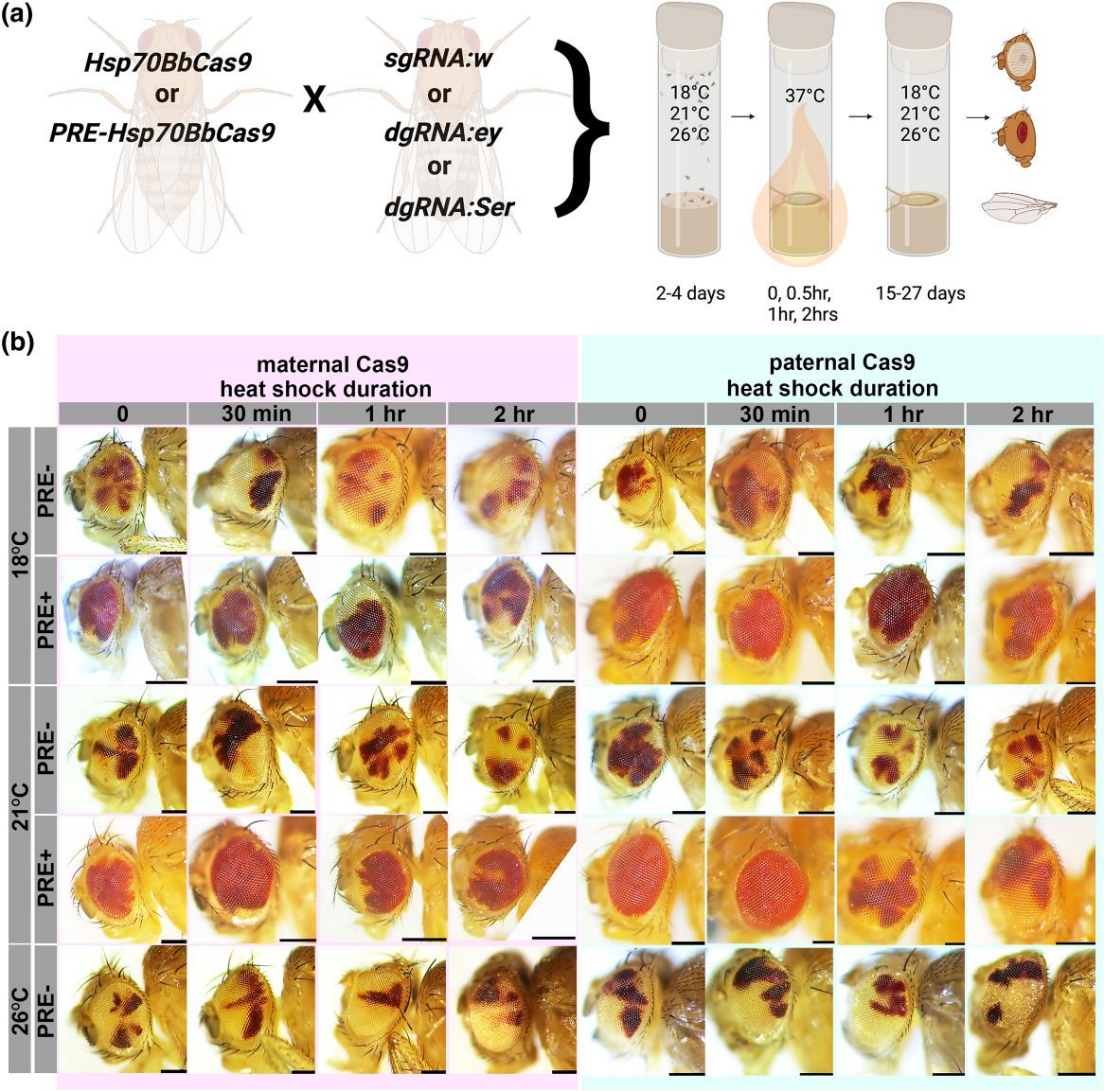
Polycomb response elements (PREs) decrease the severity of the *white* mutant phenotype. a) General schematic of genetic crosses and heat shock to generate *Cas9; gRNA* trans-heterozygotes. Created with BioRender.com. b) Representative images of *white* eye mutant phenotype variegation with and without PREs at multiple rearing temperatures (18°C, 21°C, or 26°C) and heat-shock durations (0 or no heat shock, 30 min heat shock, 1 h heat shock, or 2 h heat shock). The left four columns (highlighted in magenta) are F1 from maternal *Hsp70BbCas9* or *PRE-Hsp70BbCas9* and the right four columns (highlighted in turquoise) are F1 from paternal *Hsp70BbCas9* or *PRE-Hsp70BbCas9.* PRE + flies reared at 26°C were not scored or imaged due to suppressed *mini-white*. All scale bars = 250 μm.

**Fig. 3.**
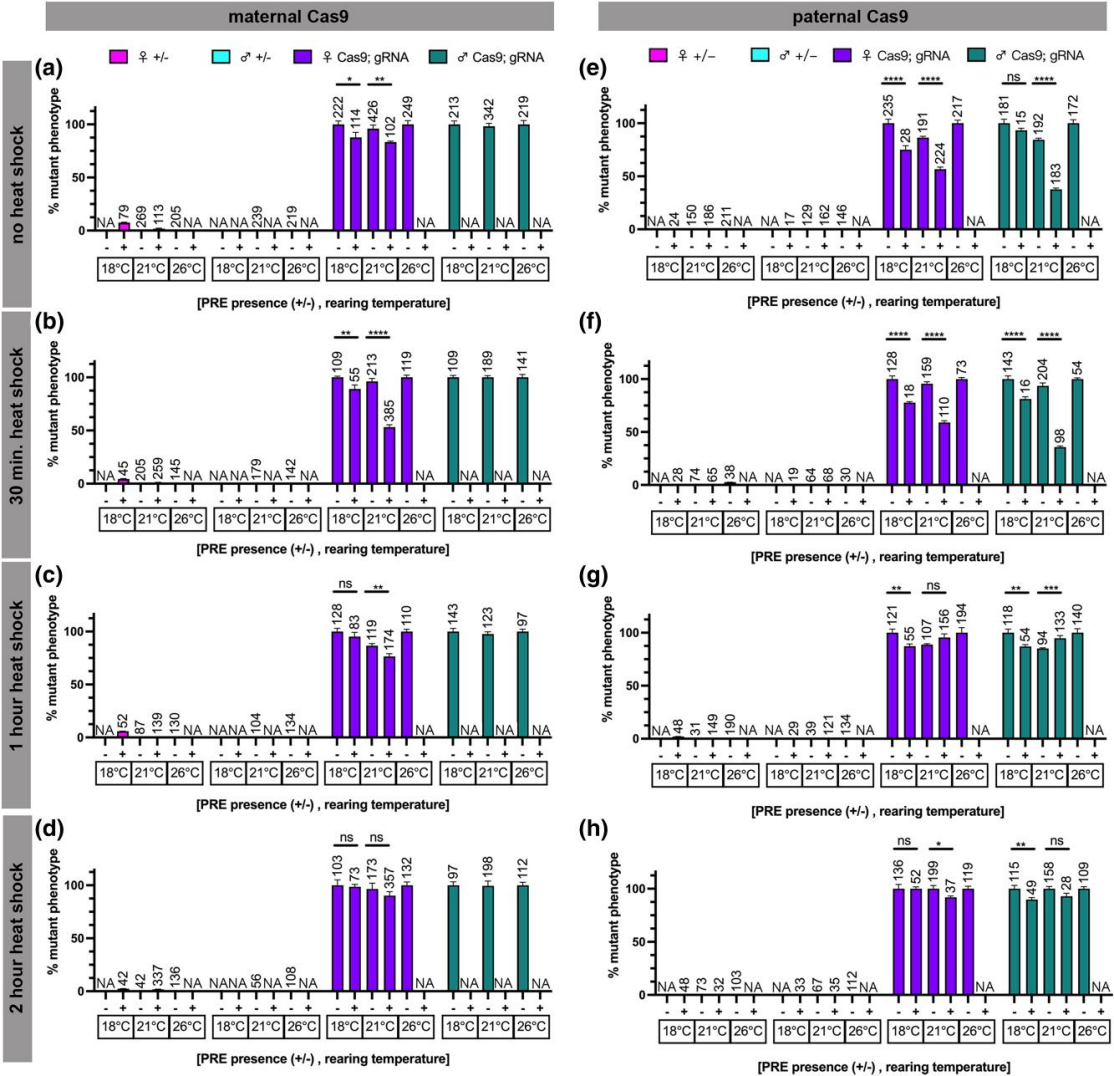
Polycomb response elements (PRE) decrease rates of Cas9-induced *white* eye phenotypes. Rate of mutant phenotype generation with heat-shock-inducible *Cas9* (*Hsp70BbCas9*) and *sgRNA:w* with and without upstream Polycomb response elements (+/− PRE, *Hsp70BbCas9* compared with *PRE-Hsp70BbCas9*), considering heat-shock duration, parental contribution, and rearing temperature. Percentage of mutant phenotype in F1 with a–d) maternal contribution of Cas9 after a) no heat-shock, b) 30 min heat-shock, c) 1 h heat-shock, d) 2 h heat-shock. Percentage of mutant phenotype in F1 with e–h) paternal contribution of Cas9 after e) no heat shock, f) 30 min heat shock, g) 1 h heat shock, and h) 2 h heat shock. Magenta = heterozygous female controls, turquoise = heterozygous male controls, purple = trans-heterozygous females, green = trans-heterozygous males. n = number of flies scored (listed above corresponding bar). Error bars = standard error of the mean. NA = not scored, see Results for details. Y-axis extends above 100% to account for error bars. Significance was not calculated for heterozygous controls. Significance was calculated using unpaired two-tailed t-test with Welch's correction. ns = *P* > 0.05, * = *P* ≤ 0.05, ** = *P* ≤ 0.01, *** = *P* ≤ 0.001, **** = *P* ≤ 0.0001.

## Results

### Construct design and *Drosophila* transgenesis

The *Cas9* (*Csn1*) endonuclease from the *Streptococcus pyogenes* Type II CRISPR/Cas system was utilized downstream of the *Drosophila melanogaster Hsp70Bb* promoter followed by a T2A sequence and enhanced GFP (eGFP) to provide heat-shock-inducible expression of *Cas9* (Kandul *et al.* 2021). *S. pyogenes Cas9* has been widely used to induce genome modification in *Drosophila melanogaster* (Bassett *et al.* 2013; Gratz *et al.* 2013, 2014; Yu *et al.* 2013; Xue *et al.* 2014) as well as many other model organisms (Wang *et al.* 2016). The *Drosophila Hsp70Bb* promoter has been widely used for inducible gene expression and has been reported to have leaky expression, where the downstream elements are expressed without incubation at the inducible heat-shock temperature (Curtin *et al.* 2008; Naidoo and Young 2012; Akmammedov *et al.* 2017). Suppression of leaky expression of *lacZ* under control of the *Hsp70Bb* promoter was achieved through the addition of upstream *bithoraxoid* (*bxd*) PREs with insulating gypsy elements (Simon *et al.* 1993; Akmammedov *et al.* 2017). Given this strong suppression of promoter activity, we sought to utilize PREs to minimize leakiness of *Cas9* under *Hsp70Bb* promoter control. The PRE repeats and gypsy insulator elements were cloned into the original Hsp70Bb-Cas9-T2A-eGFP vector and used for site-specific ɸC31 integration to generate *PRE-Hsp70BbCas9* transgenic *D. melanogaster* (see Materials and Methods and Supplementary Fig. 1) for characterization and comparison against *Hsp70BbCas9* transgenic *D. melanogaster*.

### PREs decrease relative *Cas9* transcript levels

RT-qPCR was used to assess the relative levels of *Cas9* transcripts with and without upstream PREs at multiple heat-shock durations and *D. melanogaster* rearing temperatures (Fig. 1a and b). Levels of *Cas9* transcripts were assessed relative to the housekeeping gene *Ribosomal protein L32* (*RpL32*), whose constant expression across heat-shock durations remained constant (Akmammedov *et al.* 2017) (Supplementary Fig. 2a and b).

Relative levels of *Cas9* transcripts were compared for *Hsp70BbCas9* and *PRE-Hsp70BbCas9* flies at different heat-shock durations and rearing temperatures (Fig. 1c–e). It is important to note that *Cas9* transcripts are present after no heat shock from *PRE-Hsp70BbCas9* flies (Fig. 1c and Supplementary 2d). After no heat shock, significant differences in relative *Cas9* transcript levels were found at 18°C and 21°C rearing temperatures, but not at 26°C, suggesting that higher rearing temperatures may negate any effect of the PREs on *Hsp70Bb* promoter activity (Fig. 1c–e). This suggests that PREs do not completely suppress *Hsp70BbCas9* leaky expression in this system. Average relative levels of *Cas9* transcripts generally increased with increasing heat-shock duration at all rearing temperatures for *Hsp70BbCas9* flies (Fig. 1c–e and Supplementary Fig. 2c). Relative levels of *Cas9* transcripts increased significantly in *PRE-Hsp70BbCas9* flies after all heat-shock durations at 18°C rearing (30 min = 0.0063 ± 0.0013, *P* = 0.0137; 1 h = 0.0058 ± 0.00069, *P* = 0.0048; 2 h = 0.0034 ± 0.00038, *P* = 0.0043; vs no heat shock = 0.000047 ± 0.000017) (Supplementary Fig. 2d), and after both 1 hand 2 h heat shocks at 21°C rearing (1 h = 0.0059 ± 0.00048, *P* = 0.0022; 2 h = 0.0036 ± 0.00082, *P* = 0.0176; vs no heat shock = 0.000023 ± 0.0000015) (Supplementary Fig. 2e), as well as at 26°C rearing (1 h = 0.0019 ± 0.00055, *P* = 0.0286; 2 h = 0.0012 ± 0.00033, *P* = 0.0237; vs no heat shock = 0.000034 ± 0.000014) (Supplementary Fig. 2f). Interestingly, average relative levels of *Cas9* transcripts from *PRE-Hsp70BbCas9* samples after a 2 h heat shock were lower compared with after a 1 h heat shock (Supplementary Fig. 2d–f). This may be indicative of the rapid suppression of *Cas9* expression during the 30 min recovery period after heat shock (Akmammedov *et al.* 2017).

Fold change in *Cas9* expression was calculated for these RT-qPCR data using the ΔΔCt method to assess the fold difference in *Cas9* expression in *Hsp70BbCas9* normalized to *PRE-Hsp70BbCas9* flies. Without heat shock, *Hsp70BbCas9* animals had an 85.85 ± 2.63-fold higher expression of *Cas9* at 18°C rearing temperature than *PRE-Hsp70BbCas9* animals (Supplementary Fig. 2g). As rearing temperature increased, this fold change in expression decreased (38.80 ± 12.11 for 21°C and 7.41 ± 3.90 for 26°C) (Supplementary Fig. 2g). After heat shock, the average fold change in expression exceeded 130 (133.01 ± 47.77 for 30 min heat shock, 18°C rearing, see Supplementary Fig. 2g) with the highest average fold change at 820.49 for 30 min heat shock, 21°C rearing (Supplementary Fig. 2h). After 1 h or 2 h heat shocks, no significant difference in fold change in expression of *Cas9* was observed across all rearing temperatures (Supplementary Fig. 2j and k). Revisualizing these data as fold change by heat-shock duration per rearing temperature reveals large differences in average fold change after 30 min heat shock compared with no heat shock at 21°C and 26°C rearing temperatures (Supplementary Fig. 2m and n). The largest average fold change difference for 18°C rearing was after a 2 h heat shock (Supplementary Fig. 2l). Significant differences in fold change compared with no heat-shock conditions were found for 21°C rearing after all heat-shock durations (30 min = 820.49 ± 90.54, *P* = 0.0039; 1 h = 165.54 ± 24.47, *P* = 0.0044; 2 h = 412.57 ± 82.94, *P* = 0.0145; vs no heat shock = 38.8 ± 12.11) (Supplementary Fig. 2m), and for 26°C rearing after a 2 h heat shock (550.50 ± 72.58, *P* = 0.0058; vs no heat shock = 7.41 ± 3.90) (Supplementary Fig. 2g and n). Overall, rearing *PRE-Hsp70BbCas9* flies at either 18°C or 21°C and using either a 2 h or 30 min heat shock, respectively, could allow for both minimization of leaky expression of *Cas9* without heat shock and maximization of *Cas9* expression after heat shock.

### Selection of gene targets and confirmation of mutant phenotypes

Given the significant decrease in, but not complete suppression of, *Cas9* transcripts with PREs upstream of the *Hsp70Bb* promoter, we sought to characterize to what extent these relatively low levels of Cas9 are able to induce mosaic mutant phenotypes. To further characterize the ability of the inducible promoter to generate functional levels of Cas9, *PRE-Hsp70BbCas9 D. melanogaster* transgenic line was compared with *Hsp70BbCas9* for their ability to generate F1 mutant phenotypes at different rearing temperatures (18°C, 21°C, and 26°C) and varying heat-shock durations (no heat shock, 30 min, 1 h, or 2 h) (Fig. 2a). Three genes with established guide RNA (gRNA) *D. melanogaster* stocks (*sgRNA:w, dgRNA:ey, dgRNA:Ser*) were used to characterize mutant phenotype generation (Fig. 2a and Supplementary Reagents Table). *sgRNA:w* was used since targeting the *white* gene often results in a prominent, visible phenotype of either white eyes or variegated eyes with white and red ommatidia, allowing for ease of identification while also allowing for a description of a high-efficacy gRNA. *sgRNA:ey,* targeting the *eyeless* gene also results in a prominent, visible phenotype, but was chosen given its location on the highly heterochromatic 4th, or dot, chromosome, as an example for a low-efficacy gRNA stock. *sgRNA:Ser* was chosen to target *Serrate*, a dominant gene with a visible and often lethal phenotype.

Mutant phenotypes were generated with constitutively expressed *Cas9* stocks, nanos-Cas9 (*nosCas9*) and vasa-Cas9 (*vasaCas9*), as controls. When crossed with *sgRNA:w*, *nosCas9* generated a low frequency of variegated eyes in the F1 (maternal Cas9: 5.71% of F1 females and 6.38% F1 males; paternal Cas9: 0% for both F1 females and males) compared with *vasaCas9* (maternal Cas9: 100.0 ± 0.0% of F1 females, males were not scored; paternal Cas9: 100.0% for both F1 females and males) (Supplementary Fig. 3a). *nosCas9* also generated milder phenotypes (Supplementary Fig. 3b and c) compared with *vasaCas9* (Supplementary Fig. 3d and e). When crossed with *dgRNA:ey*, *nosCas9* generated zero mutants (maternal Cas9: 0% of both F1 females and males; paternal Cas9: 0% of both F1 females and males) (Supplementary Fig. 3g) compared with *vasaCas9* (maternal Cas9: 16.67 ± 23.57% of F1 females and 33.33 ± 23.57% of F1 males; paternal Cas9: 30.0 ± 42.43% of F1 females and 28.57 ± 40.41% F1 males) (Supplementary Fig. 3f). Maternal *vasaCas9* produced slightly more mutant progeny than paternal *vasaCas9*, however, the severity of the mutant phenotype was often similar (Supplementary Fig. 3h and k) with rare full phenotypes (Supplementary Fig. 3j). When crossed with *dgRNA:Ser*, *nosCas9* was able to generate viable F1 whereas *vasaCas9* generated lethal phenotypes (Supplementary Fig. 3l). *nosCas9* was further able to generate *Serrate* mutant phenotypes when inherited maternally (3.33% of F1 females and 4.76% of F1 males) but not paternally (0% of F1 females and F1 males) (Supplementary Fig. 3l). These results provide a range of mutant rate formation, where *nosCas9* generally generated lower rates of or less severe phenotypes compared to *vasaCas9*. Simultaneously, these data allow for comparison to the rate of mutant phenotype formation generated by inducible *Cas9* expression.

### PREs reduce the severity of *white* mutant phenotype induced by *Hsp70BbCas9*

Trans-heterozygous *sgRNA:w; Cas9* F1 flies with or without PREs were scored for the presence of any *white* mutant phenotype after heat shock. The rate of *white* mutant phenotype formation in *sgRNA:w; Cas9* trans-heterozygous flies was high across all conditions (Figs. 2 and 3) (all but two conditions were over 50%), and closer to rates of mutant phenotype formation by *vasaCas9* controls (maternal Cas9: 100 ± 0% F1 females; paternal Cas9: 100% F1 females and 100% F1 males) compared with *nosCas9* controls (maternal Cas9: 5.71% F1 females and 6.38% F1 males; paternal Cas9: 0% F1 females and 0% F1 males) (Supplementary Fig. 3a). All heat shock durations at both 18°C and 26°C rearing temperatures for maternal and paternal *Hsp70BbCas9* (PRE- trans-heterozygotes) averaged 100% visible mutant phenotypes (Fig. 3). The rate of visible mutant phenotypes varied for 21°C rearing temperatures for *Hsp70BbCas9* F1 trans-heterozygous flies but did not drop below 84% (Fig. 3). The reason for this discrepancy between rearing temperatures remains unknown. These results suggest that there is no obvious or consistent difference between *Hsp70BbCas9* maternal and paternal inheritance in combination with *sgRNA:w*.

Presence of PREs upstream of the *Hsp70Bb* promoter resulted in modest, yet statistically significant decreases in rates of *white* mutant phenotypes for most conditions tested (parental Cas9 inheritance, rearing temperature, and heat-shock duration) (Fig. 3). For both maternal and paternal Cas9 inheritance, as the heat shock duration increased, differences between mutant phenotype formation in female F1 lost significance. After a 2 h heat shock, there was no significant difference in percent mutant phenotype of female F1 with maternal Cas9 with or without PREs at 18°C rearing (PRE+: 98.63 ± 2.19% vs PRE-: 100 ± 5.08%) or 21°C rearing (PRE+: 90.20 ± 3.80% vs PRE-: 96.53 ± 5.39%), nor a significant difference in percent mutant phenotype of F1 with paternal Cas9 with or without PREs at 18°C rearing for female F1 (PRE+: 100 ± 1.80% vs PRE-: 100 ± 3.97%) or at 21°C rearing for male F1 (PRE+: 92.86 ± 2.90% vs PRE-: 100 ± 2.10%) (Fig. 3). This suggests that the longer the heat-shock duration, the less effective PREs become in suppressing functional levels of *Cas9* transcripts in the presence of a highly efficacious gRNA.

Representative images of trans-heterozygous flies were collected to qualitatively evaluate the extent of *white* phenotype since the binary calls of our quantification of the rate of mutant formation does not distinguish between mild variegation (small portions of white ommatidia), severe variegation (large portions of white and red ommatidia), or highly mutated ommatidia (mostly white ommatidia). The majority of the *white* mutant trans-heterozygous flies without PREs (*sgRNA:w; Hsp70BbCas9*) (Fig. 2, rows 1, 3, and 5) show strong variegation phenotypes, regardless of parental Cas9 contribution (maternal vs paternal), rearing temperature, or heat-shock duration. Trans-heterozygous flies with PREs (*sgRNA:w; PRE-Hsp70BbCas9*) show a range of variegation phenotypes: some flies show no variegation (e.g. paternal Cas9, 21°C rearing, no heat shock and 30 min heat shock), and the longer heat-shock durations (1 h and 2 h) appear to result in increased variegation at all rearing temperatures (Fig. 2, rows 2 and 4). *sgRNA:w; PRE-Hsp70BbCas9* trans-heterozygous flies reared at 26°C were not scored due to PRE suppression of the *mini-white* transgenic marker (Kassis *et al.* 1991; Akmammedov *et al.* 2017) to avoid any uncertainty regarding the presence of the PRE-Hsp70Bb-Cas9 transgene (labeled as NA in Fig. 3). Heterozygous controls at 18°C were often not included (labeled as NA in Fig. 3); the *CyO* balancer curly wing phenotype often appeared suppressed in *CyO; Hsp70BbCas9* heterozygous F1 (not scored). This suppressed curly wing phenotype is most likely due to reduced expression of *Curly* (*Cy*) at temperatures below 19°C (Pavelka *et al.* 1996). Given the difficulty to distinguish between heterozygous and trans-heterozygous F1 flies, only homozygous *sgRNA:w* P0 were used to generate trans-heterozygous *sgRNA:w; Hsp70BbCas9* flies at 18°C. Finally, *sgRNA:w; PRE-Hsp70BbCas9* trans-heterozygous males with maternally inherited Cas9 were not scored due to difficulty scoring the low contrasted (and PRE-suppressed) *mini-white* transgene variegation in a *white* mutant background (Kassis *et al.* 1991; Akmammedov *et al.* 2017). Overall, PREs upstream of *Hsp70BbCas9* are able to decrease the severity of *white* mutant variegation for most conditions tested.

Individual trans-heterozygous *sgRNA:w; Hsp70BbCas9* flies were genotyped and compared to heterozygous controls to identify the presence of targeted DNA mutations by Cas9. Since F1 from *sgRNA:w* crosses were heterogeneous for *white* alleles which cannot be separated by Sanger sequencing, the *mini-white* transgene was sequenced for genotyping purposes. Cas9 was able to induce DNA mutations in trans-heterozygous *sgRNA:w; Hsp70BbCas9* flies with mutant phenotypes when inherited maternally (Supplementary Fig. 4c) or paternally (Supplementary Fig. 4e), suggesting that scoring mutant phenotype rate can act as a proxy for Cas9 function.

### PREs upstream *Hsp70BbCas9* reduce the efficacy of already low-efficiency guide RNAs targeting *eyeless*

**Fig. 4.**
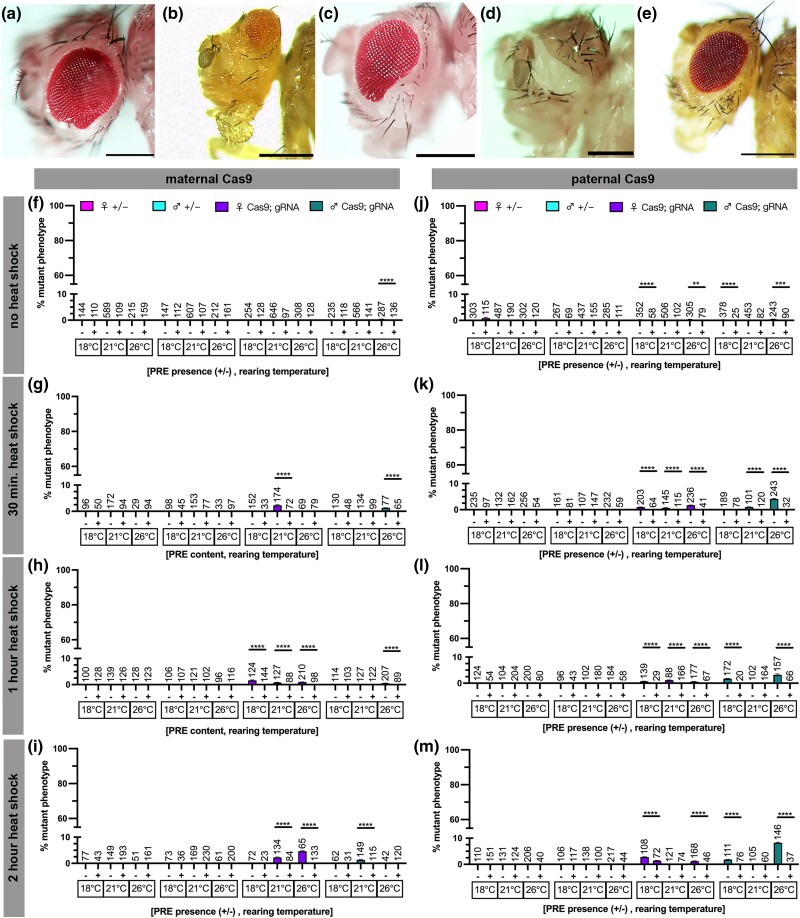
PREs further reduce a rare eyeless phenotype. a–e) Representative images of the phenotypic range of induced *eyeless* phenotypes with a, b) maternal *Hsp70BbCas9*, c, d) paternal *Hsp70BbCas9*, and e) maternal *PRE-Hsp70BbCas9*. All scale bars = 250 μm. f–m) Rate of mutant phenotype generation with heat-shock-inducible *Cas9* (*Hsp70BbCas9*) and *dgRNA:ey* with and without upstream Polycomb response elements (+/− PRE), considering heat-shock duration, Cas9 contribution, and rearing temperature. Percentage of mutant phenotype in F1 with f–i) maternal contribution of Cas9 after f) no heat shock, g) 30 min heat shock, h) 1 h heat shock, i) 2 h heat shock. Percentage of mutant phenotype in F1 with j–m) paternal contribution of Cas9 after j) no heat shock, k) 30 min heat shock, l) 1 h heat shock, and m) 2 h heat shock. The Y-axis was modified to visualize lower values while also maintaining 100% the maximum value. Magenta = heterozygous female controls, turquoise = heterozygous male controls, purple = trans-heterozygous females, green = trans-heterozygous males. n = number of flies scored (listed above corresponding bar). Error bars = standard error of the mean. Significance was not calculated for heterozygous controls. Unlabeled = 0 ± 0% for both values (and no significance calculated). Significance was calculated using unpaired two-tailed *t*-test with Welch's correction. ** = *P* ≤ 0.01, *** = *P* ≤ 0.001, **** = *P* ≤ 0.0001.

The rate of *eyeless* (*ey*) mutant phenotype formation was very low across all conditions (Fig. 4f–n) (under 10%), consistent with low mutant phenotype formation with constitutive *nosCas9* and *vasaCas9* controls (Supplementary Fig. 3g). Rearing temperature did not change the low rate of *ey* mutant phenotype formation with no heat shock (Fig. 4f and k). All mutant phenotype rates were relatively low with the highest rate of mutant phenotype formation seen at 26°C rearing temperature for F1 females inheriting maternal *Hsp70BbCas9* (4.62 ± 0.18%) and F1 males inheriting paternal *Hsp70BbCas9* (8.22 ± 0.23%) both after a 2 h heat shock (Fig. 4f–n). The majority of heat-shock-induced *ey* phenotypes observed were mild, similar to Fig. 4a, c and e; on rare occasions a large portion or the majority of the eye would not develop, as in Fig. 4b and d (severity of mutant phenotype not quantified). Regardless of low mutant phenotype generation, presence of PREs significantly decreased the rate of mutant phenotype in F1 in every condition where mutants were identified (Fig. 4f–n). In fact, *ey* mutants were only identified in *dgRNA:ey; PRE-Hsp70BbCas9* trans-heterozygous F1 under one condition (female F1 at 18°C rearing and paternal Cas9: 1.39 ± 0.13%) (Fig. 4k).

Individual flies were genotyped using Sanger sequencing to identify the presence of targeted DNA mutations by Cas9 in flies with or without a mutant phenotype. All individuals sequenced showed no DNA modifications compared with controls for *ey* gRNA target 1 (Supplementary Fig. 5, left column). Regardless of phenotype status, trans-heterozygous individuals sequenced showed low levels of secondary peaks adjacent to the *ey* gRNA target 2 site (Supplementary Fig. 5d, f, h and k, right column) compared with most heterozygous controls (Supplementary Fig. 5c, e, g and j). Paternal *Hsp70BbCas9* heterozygous control however revealed similar low levels of secondary peaks (Supplementary Fig. 5e). The reason for this subtle sequence difference remains unclear, however, given the low rates of mutant phenotype generation and low severity of mutant phenotypes suggests that the majority of DNA sequences present may have been wild type, obscuring any mutant genotype. We suspect that PCR amplicons of the wild type sequences were in high abundance compared to mutant sequences given that sequencing of individual F1 from *vasaCas9* crosses revealed multiple peaks at both *ey* gRNA target 1 and gRNA target 2 sites (Supplementary Fig. 6e and g).

### PREs upstream of *Hsp70BbCas9* allow for the generation of viable *Serrate* mutants

**Fig. 5.**
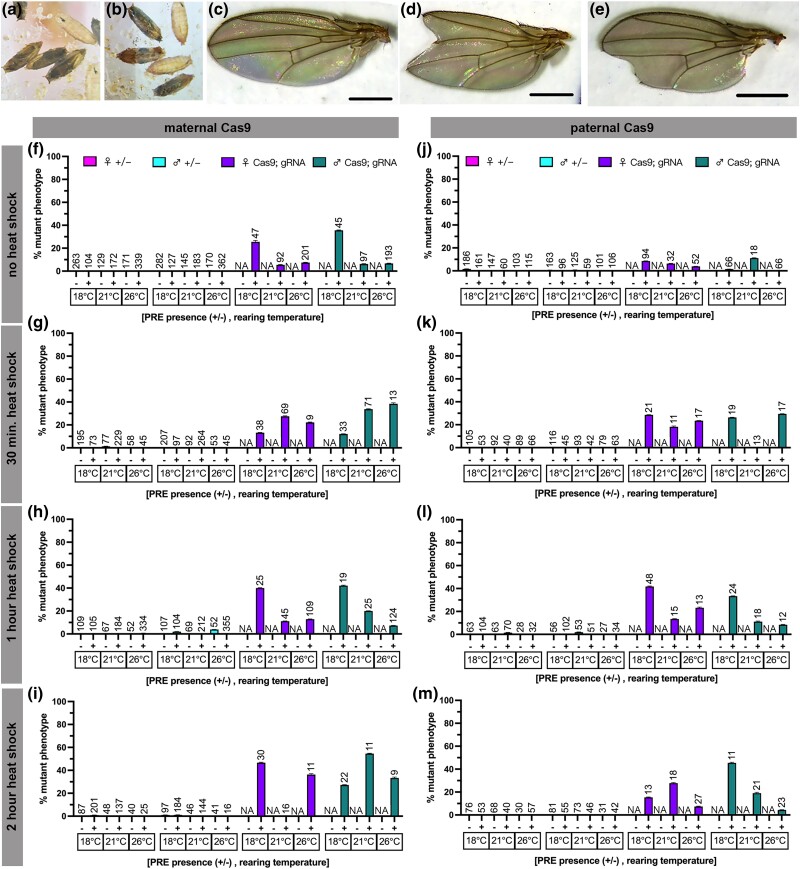
PRE elements allow for viable *Serrate* phenotypes in an otherwise lethal genotype. Representative images of induced *Serrate* mutant phenotypes with *dgRNA:Ser*. a, b) Lethal pupae from a) maternal *Hsp70BbCas9,* and b) paternal *Hsp70BbCas9*. c–e) *PRE-Hsp70BbCas9*/*dgRNA:Ser* trans-heterozygotes wing examples with maternal *PRE-Hsp70BbCas9* at 26°C rearing with no heat shock C) with no serration, and d, e) with serration. All scale bars = 500 μm. Percentage of mutant phenotype generation with heat-shock-inducible *Cas9* (*Hsp70BbCas9*) and *dgRNA:Ser* with and without upstream Polycomb response elements (+/− PRE), considering heat-shock duration, Cas9 contribution, and rearing temperature. f–i) Maternal contribution of Cas9 after f) no heat shock, g) 30 min heat shock, h) 1 h heat shock, i) 2 h heat shock. j–m) Paternal contribution of Cas9 after j) no heat shock, k) 30 min heat shock, l) 1 h heat shock, and m) 2 h heat shock. Magenta = heterozygous female controls, turquoise = heterozygous male controls, purple = trans-heterozygous females, green = trans-heterozygous males. n = number of flies scored (listed above corresponding bar). Error bars = standard error of the mean. Significance was not calculated for heterozygous controls. Significance was not calculated for trans-heterozygous flies since all *Hsp70BbCas9*/*dgRNA:Ser* (PRE -) flies died during metamorphosis.

Mutations of *Serrate* by *Hsp70BbCas9* resulted in complete lethality (100%) of trans-heterozygous flies regardless of heat-shock duration or rearing temperature (Fig. 5f–n). All trans-heterozygous flies appeared to die during pupal development (Fig. 5a and b, unscored). Individual lethal pupae were genotyped using Sanger sequencing and found to have sequence mutations at either *Ser* gRNA target 1 or gRNA target 2 sites compared with adult heterozygous controls (Supplementary Fig. 7). Addition of PREs allowed for viable trans-heterozygotes and allowed for formation of visible mutant phenotypes (Fig. 5d and e) for most conditions (Fig. 5f–n). Exactly why this lethality was rescued with the addition of PREs, whether due to decreases in off-target effects, changes in mutation rate, or changes in mutation type, remains for future studies.

To verify DNA mutations in viable trans-heterozygotes, individual *dgRNA:Ser; PRE-Hsp70BbCas9* flies with or without a visible phenotype were genotyped by Sanger sequencing (Supplementary Fig. 8). Individual F1 with maternal *PRE-Hsp70BbCas9* without serrated wings, revealed nucleotide changes only at gRNA target 1 (Supplementary Fig. 8d), suggesting either differences in the severity of different mutant alleles, or insufficient mosaicism to induce a visible phenotype. F1 with maternal *PRE-Hsp70BbCas9* with serrated wings revealed nucleotide changes at both gRNA targets (Supplementary Fig. 8e). Individual F1 with paternal *PRE-Hsp70BbCas9* showed no nucleotide changes at both gRNA target sites regardless of the presence of a visible serrated wing (Supplementary Fig. 8g and h). This either suggests that paternal *PRE-Hsp70BbCas9* resulted in fewer mutant cells in mosaic animals compared with maternal Cas9, off-target effects from mutating genes related to wing morphology such as *Additional Sex Combs* or false positives (general wing damage) were scored as *Serrate* mutants (Fig. 5, see heterozygous control values).

Pupal lethality of *dgRNA:Ser; vasaCas9* trans-heterozygotes corresponded to sequence differences compared with reference sequences around gRNA target 1 and/or 2 for both maternal (Supplementary Fig. 9g and l, respectively) and paternal *vasaCas9* (Supplementary Fig. 9h and m, respectively). Sanger sequencing of *dgRNA:Ser; nosCas9* trans-heterozygotes revealed a large deletion with maternal *nosCas9* (Supplementary Fig. 9c) compared with reference sequences, corresponding to the reported visible mutant phenotype (3.33% of F1 females and 4.76% of F1 males) (Supplementary Fig. 3l). No nucleotide differences were observed for paternal *nosCas9* (Supplementary Fig. 9d) corresponding to a rate of 0% mutant phenotype formation (Supplementary Fig. 3l).

## Discussion

### PREs significantly reduce leakiness of *Cas9* without heat shock

Here we characterize the ability of PREs to suppress heat-shock-induced *Cas9* expression and indirectly, the ability to limit Cas9 functionality. Our results corroborate previous reports characterizing the ability of PREs to significantly suppress leaky expression of the *Drosophila Hsp70Bb* promoter (Akmammedov *et al.* 2017). We further characterize PREs in the context of inducible Cas9-directed genome editing. Despite seeing significant decreases in relative *Cas9* transcript levels, we are able to induce genome modifications with PREs upstream of the *Hsp70Bb* promoter when flies were reared at 18°C in the absence of a heat shock. This suggests that low levels of *Cas9* transcripts are sufficient to induce genome modifications and produce mutant mosaic phenotypes. Further modifications, either more PREs or in combination with temperature-sensitive inteins (Zeidler *et al.* 2004), are needed to completely suppress *Hsp70Bb* promoter activity with no heat shock to allow for precise control of expression of downstream genes.

### Many variables influence the effectiveness of PREs to reduce Cas9 efficacy with a heat-shock-inducible promoter

Characterizing *PRE*-*Hsp70BbCas9* with gRNAs targeting three separate genes revealed that the gene target and/or the gRNAs themselves impact the downstream effect of a functional Cas9/gRNA complex, a common occurrence in studies of genome modification with Cas9. The very high rates (up to 100%) of biallelic mosaicism, by crossing Cas9 to *sgRNA:w* producing a clear visible phenotype, and lethal biallelic mosaicism by crossing to *dgRNA:Ser*, reveal this difference in comparison to *dgRNA:ey* which resulted in much lower rates of biallelic mosaicism in the form of a visible phenotype. Whether or not these differences in rate of visible biallelic mosaicism corresponds to different spatial expression levels of Cas9 mRNA and/or protein remains unknown. Effectiveness of PREs to reduce leaky expression of *Hsp70Bb* promoter is known to be influenced by genome integration site, proximal elements of the integration site, and tissue- and development-specific influences on Polycomb-based silencing (Dellino *et al.* 2004; Steffen and Ringrose 2014; Akmammedov *et al.* 2017). When generating *PRE-Hsp70BbCas9* systems, characterizing multiple transgene integration sites may aid in developing less-leaky inducible gene systems.

*D. melanogaster* rearing temperature appears to minimally affect levels of mutant formation by *Hsp70BbCas9* (without PREs) for efficacious gRNAs (*sgRNA:w* and *dgRNA:Ser*) at all heat-shock durations. High rearing temperatures combined with long heat shocks appear to increase the rate of mutant phenotype for low efficient gRNA stocks (*dgRNA:ey*) without PREs, in our hands. Presence of PREs in *PRE-Hsp70BbCas9* allowed for more nuanced changes in rates of mutant phenotype formation for efficacious gRNAs (*sgRNA:w* and *dgRNA:Ser*). Higher rearing temperatures (21°C) for *PRE-Hsp70BbCas9* resulted in lower rates of *white* mutant phenotype formation compared with at 18°C after either no heat shock or 30 min heat shocks (Fig. 3). Often for *Serrate* mutant phenotype formation, higher rearing temperatures (26°C) with longer heat-shock durations (1 h or 2 h) resulted in lower rates of visible biallelic mosaicism (Fig. 5h, j, m and n). Whether these numbers are a result of an increase in lethality remains unknown. Rearing temperature may be an important factor depending on the gene target and/or gRNA and details regarding whether or not these differences seen at varying rearing temperatures are due to differences in Cas9 function or PRE silencing ability remain for future studies.

### Using PREs in combination with genetic pest control strategies

A non-leaky, inducible promoter could be useful for generating gene drives (Champer *et al.* 2016) or other Cas9 modified fertile animals for large rearing and release while limiting the amount of Cas9 present in released animals. Lower levels of Cas9 RNA and protein may limit detrimental effects of Cas9 spreading through wild populations of pest species, depending on the gRNA and gene target, but this requires further exploration and detailed characterization for each gene drive system designed. Using CRISPR/Cas9 for sterile insect technique (SIT), as in precision-guided SIT (pgSIT), alleviates the concern for population spread of Cas9 since every released transgenic animal is sterile and therefore unable to pass on the transgene of concern (Kandul *et al.* 2019, 2021, 2022). A non-leaky temperature-inducible pgSIT (TI-pgSIT) would allow for chromosomal linkage of Cas9 and gRNAs without collapse of the transgenic stock population. This chromosomally linked TI-pgSIT stock would further eliminate the need to collect flies from separate strains; embryos could be heat shocked enmasse to generate lethal females and sterile males for release and allow for effective pest population control (Kandul *et al.* 2019, 2021).

## Supplementary Material

jkad024_Supplementary_Data

## Data Availability

Strains are available through Bloomington *Drosophila* Stock Center (BDSC). Plasmids are available through Addgene. The Supplementary Reagents Table contains details of all reagents used in this study. Supplemental material available at G3 online.

## References

[jkad024-B1] Akmammedov A , Geigges M, Paro R. Single vector non-leaky gene expression system for Drosophila melanogaster. Sci Rep. 2017;7(1):6899. doi:10.1038/s41598-017-07282-w. 28761084 PMC5537222

[jkad024-B2] Bassett AR , Tibbit C, Ponting CP, Liu J-L. Highly efficient targeted mutagenesis of Drosophila with the CRISPR/Cas9 system. Cell Rep. 2013;4(1):220–228. doi:10.1016/j.celrep.2013.06.020. 23827738 PMC3714591

[jkad024-B3] Beisel C , Paro R. Silencing chromatin: comparing modes and mechanisms. Nat Rev Genet. 2011;12(2):123–135. doi:10.1038/nrg2932. 21221116

[jkad024-B4] Champer J , Buchman A, Akbari OS. Cheating evolution: engineering gene drives to manipulate the fate of wild populations. Nat Rev Genet. 2016;17(3):146–159. doi:10.1038/nrg.2015.34. 26875679

[jkad024-B5] Corces V , Pellicer A, Axel R, Meselson M. Integration, transcription, and control of a drosophila heat shock gene in mouse cells. Proc Natl Acad Sci U S A. 1981;78(11):7038–7042. doi:10.1073/pnas.78.11.7038. 6273904 PMC349189

[jkad024-B6] Curtin JF , Candolfi M, Xiong W, Lowenstein PR, Castro MG. Turning the gene tap off; implications of regulating gene expression for cancer therapeutics. Mol Cancer Ther. 2008;7(3):439–448. doi:10.1158/1535-7163.MCT-07-2328. 18347132 PMC2593107

[jkad024-B7] Dellino GI , Schwartz YB, Farkas G, McCabe D, Elgin SCR, Pirrotta V. Polycomb silencing blocks transcription initiation. Mol Cell. 2004;13(6):887–893. doi:10.1016/S1097-2765(04)00128-5. 15053881

[jkad024-B8] Fujita T , Chen MJ, Li B, Smith NA, Peng W, Sun W, Toner MJ, Kress BT, Wang L, Benraiss A, et al Neuronal transgene expression in dominant-negative SNARE mice. J Neurosci. 2014;34(50):16594–16604. doi:10.1523/JNEUROSCI.2585-14.2014. 25505312 PMC4261088

[jkad024-B9] Gibson DG , Young L, Chuang R-Y, Venter JC, Hutchison CA 3rd, Smith HO. Enzymatic assembly of DNA molecules up to several hundred kilobases. Nat Methods. 2009;6(5):343–345. doi:10.1038/nmeth.1318. 19363495

[jkad024-B10] Gratz SJ , Cummings AM, Nguyen JN, Hamm DC, Donohue LK, Harrison MM, Wildonger J, O'Connor-Giles KM. Genome engineering of Drosophila with the CRISPR RNA-guided Cas9 nuclease. Genetics. 2013;194(4):1029–1035. doi:10.1534/genetics.113.152710. 23709638 PMC3730909

[jkad024-B11] Gratz SJ , Ukken FP, Rubinstein CD, Thiede G, Donohue LK, Cummings AM, O'Connor-Giles KM. Highly specific and efficient CRISPR/Cas9-catalyzed homology-directed repair in drosophila. Genetics. 2014;196(4):961–971. doi:10.1534/genetics.113.160713. 24478335 PMC3982687

[jkad024-B12] Kandul NP , Liu J, Akbari OS. Temperature-Inducible precision-guided Sterile insect technique. CRISPR J. 2021;4(6):822–835. doi: 10.1089/crispr.2021.0077. 34735288 PMC8742271

[jkad024-B13] Kandul NP , Liu J, Buchman A, Shriner IC, Corder RM, Warsinger-Pepe N, Yang T, Yadav AK, Scott MJ, Marshall JM, et al Precision guided Sterile males suppress populations of an invasive crop pest. GEN Biotechnol. 2022;1(4):372–385. doi:10.1089/genbio.2022.0019.

[jkad024-B14] Kandul NP , Liu J, Sanchez C HM, Wu SL, Marshall JM, Akbari OS. Transforming insect population control with precision guided sterile males with demonstration in flies. Nat Commun. 2019;10(1):84. doi:10.1038/s41467-018-07964-7. 30622266 PMC6325135

[jkad024-B15] Kassis JA , VanSickle EP, Sensabaugh SM. A fragment of engrailed regulatory DNA can mediate transvection of the white gene in Drosophila. Genetics. 1991;128(4):751–761. doi:10.1093/genetics/128.4.751. 1655566 PMC1204549

[jkad024-B16] Kust N , Rybalkina E, Mertsalov I, Savchenko E, Revishchin A, Pavlova G. Functional analysis of Drosophila HSP70 promoter with different HSE numbers in human cells. PLoS One. 2014;9(8):e101994. doi:10.1371/journal.pone.0101994. PMC4125163 25101947

[jkad024-B17] Larkin A , Marygold SJ, Antonazzo G, Attrill H, dos Santos G, Garapati PV, Goodman JL, Gramates LS, Millburn G, Strelets VB, et al Flybase: updates to the *Drosophila melanogaster* knowledge base. Nucleic Acids Res. 2021;49(D1):D899–D907. doi:10.1093/nar/gkaa1026. 33219682 PMC7779046

[jkad024-B18] Lindquist S . Heat shock–a comparison of Drosophila and yeast. J Embryol Exp Morphol. 1984;83 Suppl:147–161. 6398351

[jkad024-B19] Naidoo J , Young D. Gene regulation systems for gene therapy applications in the central nervous system. Neurol Res Int. 2012;2012:595410. doi:10.1155/2012/595410. PMC3261487 22272373

[jkad024-B20] Nandy S , Pathak B, Zhao S, Srivastava V. Heat-shock-inducible CRISPR/Cas9 system generates heritable mutations in rice. Plant Direct. 2019;3(5):e00145. doi:10.1002/pld3.145. PMC6603394 31404128

[jkad024-B21] Pavelka J , Kulikov AM, Marec F. Suppression of the Drosophila curly mutation by fluorescent light. Hereditas. 1996;124(2):191–197. doi:10.1111/j.1601-5223.1996.t01-1-00191.x. 8782435

[jkad024-B22] Pelham HR . A regulatory upstream promoter element in the Drosophila hsp 70 heat-shock gene. Cell. 1982;30(2):517–528. doi:10.1016/0092-8674(82)90249-5. 6814763

[jkad024-B23] Pelham HR , Bienz M. A synthetic heat-shock promoter element confers heat-inducibility on the herpes simplex virus thymidine kinase gene. EMBO J. 1982;1(11):1473–1477. doi:10.1002/j.1460-2075.1982.tb01340.x. 6327271 PMC553234

[jkad024-B24] Simon J , Chiang A, Bender W, Shimell MJ, O’Connor M. Elements of the Drosophila bithorax complex that mediate repression by polycomb group products. Dev Biol. 1993;158(1):131–144. doi:10.1006/dbio.1993.1174. 8101171

[jkad024-B25] Sloan SA , Barres BA. Looks can be deceiving: reconsidering the evidence for gliotransmission. Neuron. 2014;84(6):1112–1115. doi:10.1016/j.neuron.2014.12.003. 25521372 PMC4433290

[jkad024-B26] Spena A , Hain R, Ziervogel U, Saedler H, Schell J. Construction of a heat-inducible gene for plants. Demonstration of heat-inducible activity of the Drosophila hsp70 promoter in plants. EMBO J. 1985;4(11):2739–2743. doi:10.1002/j.1460-2075.1985.tb03997.x. 16453638 PMC554572

[jkad024-B27] Steffen PA , Ringrose L. What are memories made of? How polycomb and trithorax proteins mediate epigenetic memory. Nat Rev Mol Cell Biol. 2014;15(5):340–356. doi:10.1038/nrm3789. 24755934

[jkad024-B28] Wang H , La Russa M, Qi LS. CRISPR/Cas9 in genome editing and beyond. Annu Rev Biochem. 2016;85(1):227–264. doi:10.1146/annurev-biochem-060815-014607. 27145843 PMC13384723

[jkad024-B29] Wei R , Wilkinson H, Pfeifer K, Schneider C, Young R, Guarente L. Two or more copies of Drosophila heat shock consensus sequence serve to activate transcription in yeast. Nucleic Acids Res. 1986;14(20):8183–8188. doi:10.1093/nar/14.20.8183. 3095794 PMC311843

[jkad024-B30] Xue Z , Ren M, Wu M, Dai J, Rong YS, Gao G. Efficient gene knock-out and knock-in with transgenic Cas9 in Drosophila. G3 (Bethesda). 2014;3(4):925–929. doi: 10.1534/g3.114.010496 PMC4025491 24657904

[jkad024-B31] Yu Z , Ren M, Wang Z, Zhang B, Rong YS, Jiao R, Gao G. Highly efficient genome modifications mediated by CRISPR/Cas9 in Drosophila. Genetics. 2013;195(1):289–291. doi:10.1534/genetics.113.153825. 23833182 PMC3761309

[jkad024-B32] Zeidler MP , Tan C, Bellaiche Y, Cherry S, Häder S, Gayko U, Perrimon N. Temperature-sensitive control of protein activity by conditionally splicing inteins. Nat Biotechnol. 2004;22(7):871–876. doi:10.1038/nbt979. 15184905

