# Supplementary material for: Polycomb response elements reduce leaky expression of Cas9 under temperature-inducible Hsp70Bb promoter in Drosophila melanogaster
Source: G3 (Bethesda). 2023 Jan 27;13(4):jkad024. doi: 10.1093/g3journal/jkad024 (PMC10085756; doi:10.1093/g3journal/jkad024)
Supplement: jkad024_Supplementary_Data [file jkad024_supplementary_data.zip › Figure_S6_G3-2022-403941.docx]

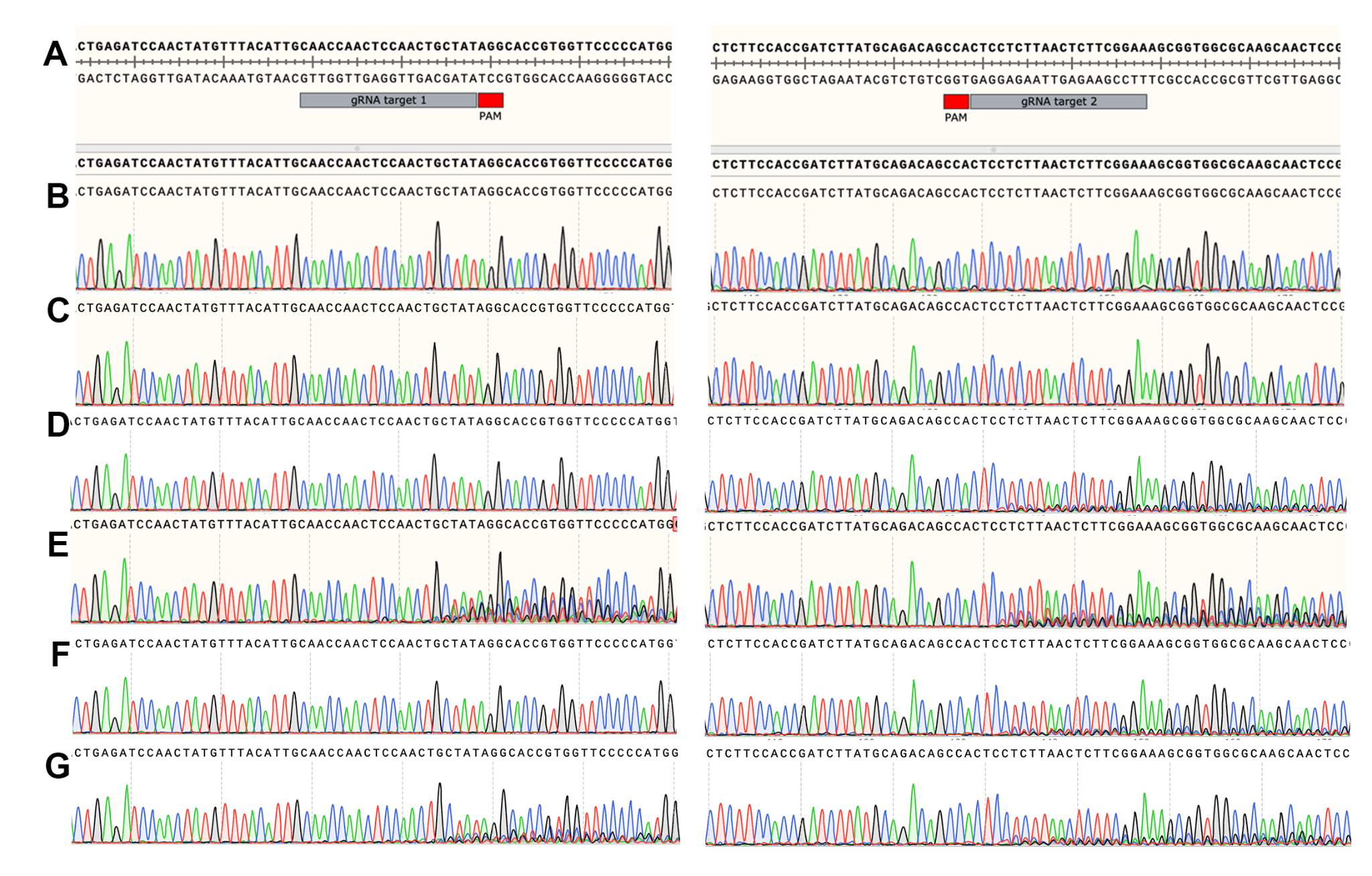


**Supplemental Figure S6. Sanger sequencing genotyping of *nanos* or *vasa* promoter-driven Cas9 targeting of *eyeless*.**
