# Supplementary material for: Polycomb response elements reduce leaky expression of Cas9 under temperature-inducible Hsp70Bb promoter in Drosophila melanogaster
Source: G3 (Bethesda). 2023 Jan 27;13(4):jkad024. doi: 10.1093/g3journal/jkad024 (PMC10085756; doi:10.1093/g3journal/jkad024)
Supplement: jkad024_Supplementary_Data [file jkad024_supplementary_data.zip › Supplemental_Material_Legends_G3-2022-403941.docx]

**Supplemental Material**

**Supplemental Figure S1. Transgene maps to compare *Hsp70BbCas9* and *PRE-Hsp70BbCas9.*** A) Hsp70Bb-Cas9-T2A-eGFP (part of Addgene 153284) used to generate *Hsp70BbCas9* transgenic *D. melanogaster*. B) PRE-Hsp70Bb-Cas9_1.3 (part of Addgene 190797) used to generate *PRE-Hsp70BbCas9* transgenic *D. melanogaster*. Differences between the transgenes are highlighted in yellow and consist of the Polycomb response elements (PREs) (neon green) and gypsy insulator elements (teal). Both transgenes were inserted using ɸC31-mediated integration into the same genomic location (see Materials and Methods).

**Supplemental Figure S2. Effects of Polycomb response elements on relative *Cas9* transcript levels.** Standardization of A) *RpL32* primers and B) *Cas9* primers. C) Relative ratio (E*_RpL32_*^RpL32_Ct^/E*_Cas9_*^Cas9_Ct^) of *Cas9*/*RpL32* transcripts at varying rearing temperatures and heat shock durations for C) *Hsp70BbCas9* (only 26°C shown) and D-F) *PRE-Hsp70BbCas9*. Data from Figure 1C-E re-graphed here for visualization purposes. Red = *Hsp70BbCas9*, blue = *PRE-Hsp70BbCas9*. Error bars = standard deviation. Significance calculated using unpaired two-tailed t-test with Welch’s correction. ns = P > 0.05, * = P ≤ 0.05 ** = P ≤ 0.01, *** = P ≤ 0.001, **** = P ≤ 0.0001. Statistics for C-F were calculated for each heat shock duration (30 minute, 1 hour, 2 hour) compared to no heat shock. Please note differences in y-axis scaling. Normalized expression ratio (fold change, calculated using ΔΔCt method) of *Cas9* transcripts for *Hsp70BbCas9* : *PRE-Hsp70BbCas9* (Hsp:PRE) plotted against G-K) rearing temperature (per heat shock duration) and against L-N) heat shock duration (per rearing temperature). G-K and L-N are the same data, replotted for visualization purposes. Error bars = standard deviation. Significance was calculated using unpaired two-tailed t-test with Welch’s correction. ns (or unlabeled) = P > 0.05, * = P ≤ 0.05, ** = P ≤ 0.01, **** = P ≤ 0.0001. Statistics for L-N were calculated for heat shock duration (30 minutes, 1 hour, 2 hours) compared to no heat shock.

**Supplemental Figure S3. Mutant phenotype formation induced by either *nanosCas9* or *vasaCas9* controls.** Quantification of F1 mutant phenotypes for A) *sgRNA:w*, F) *dgRNA: ey*, and L) *dgRNA:Ser* with either *nosCas9* or *vasaCas9* inherited maternally or paternally. *vasaCas9/dgRNA:Ser* trans-heterozygous flies (maternal and paternal Cas9) all died during pupal development and were not scored/reported in this figure. Purple = female trans-heterozygous, green = male trans-heterozygous. n = number of flies scored (listed above corresponding bar). Error bars = standard deviation. Significance was not calculated. B-E) Representative images of the phenotypic range of Cas9-induced *white* phenotypes with B) maternal *nosCas9*, C) paternal *nosCas9*, D) maternal *vasaCas9*, E) paternal *vasaCas9*. G-K) Representative images of the phenotypic range of Cas9-induced *eyeless* phenotypes with G) paternal *nosCas9*, H) maternal *vasaCas9*, J-K) paternal *vasaCas9*. All scale bars = 250μm.

**Supplemental Figure S4. Sanger sequencing genotyping of heat-shock-induced Cas9 targeting of *mini-white*.** F1 generated from *Hsp70BbCas9* crossed with *sgRNA:w* were genotyped for mutations at the *mini-white* transgene since the F1 had heterogenous *white* alleles which Sanger sequencing cannot distinguish. F1 from *PRE-Hsp70BbCas9* crosses were not genotyped. A) Transgene sequence from plasmid Hsp70Bb-Cas9-T2A-eGFP, B) maternal *Hsp70BbCas9* heterozygous control, C) maternal *Hsp70BbCas9* trans-heterozygous with a phenotype, D) paternal *Hsp70BbCas9* heterozygous control, E) paternal *Hsp70BbCas9* trans-heterozygous with a phenotype.

**Supplemental Figure S5. Sanger sequencing genotyping of heat-shock-induced Cas9 targeting of *eyeless*.** Left column = chromatograms of gRNA target 1 sequence. Right column = chromatograms of gRNA target 2 sequence. A) Reference *D. melanogaster* genome sequence of *eyeless* from NCBI Reference Sequence: NC_004353.4, B) *w^1118^*, C) maternal *Hsp70BbCas9* heterozygous control, D) maternal *Hsp70BbCas9* trans-heterozygous with a phenotype, E) paternal *Hsp70BbCas9* heterozygous control, F) paternal *Hsp70BbCas9* trans-heterozygous with a phenotype, G) maternal *PRE-Hsp70BbCas9* heterozygous control, H) maternal *PRE-Hsp70BbCas9* trans-heterozygous without a phenotype, J) paternal *PRE-Hsp70BbCas9* heterozygous control, K) paternal *PRE-Hsp70BbCas9* trans-heterozygous with a phenotype.

**Supplemental Figure S6. Sanger sequencing genotyping of *nanos* or *vasa* promoter-driven Cas9 targeting of *eyeless*.** Left column = chromatograms of gRNA target 1 sequence. Right column = chromatograms of gRNA target 2 sequence. A) Reference *D. melanogaster* genome sequence of *eyeless* from NCBI Reference Sequence: NC_004353.4, B) *w^1118^*, C) F1 from ♀*nosCas9* x ♂*dgRNA:ey* without a phenotype, D) F1 from ♀*dgRNA:ey* x ♂*nosCas9* without a phenotype, E) F1 from ♀*vasaCas9* x ♂*dgRNA:ey* with a phenotype, F) F1 heterozygous control from ♀*dgRNA:ey* x *vasaCas9*, G) F1 from ♀*dgRNA:ey* x *vasaCas9* with a phenotype.

**Supplemental Figure S7. Sanger sequencing of heat-shock induced *Hsp70BbCas9* targeting of *Serrate.***  A) Reference *D. melanogaster* genome sequence of *Serrate* from NCBI Reference Sequence: NT_033777.3. gRNA target sequences are annotated in gray boxes, PAMs annotated in red boxes. Sequencing calls and corresponding chromatograms for B) *w^1118^*, C) maternal *Hsp70BbCas9* heterozygous control, D) lethal pupae with maternal *Hsp70BbCas9*, E) paternal *Hsp70BbCas9* heterozygous control, F) lethal pupae with paternal *Hsp70BbCas9*.

**Supplemental Figure S8. Sanger sequencing of heat-shock induced *PRE*-*Hsp70BbCas9* targeting of *Serrate.*** A) Reference *D. melanogaster* genome sequence of *Serrate* from NCBI Reference Sequence: NT_033777.3. gRNA target sequences are annotated in gray boxes, PAMs annotated in red boxes. Sequencing calls and corresponding chromatograms for B) *w^1118^*, C) maternal *PRE-Hsp70BbCas9* heterozygous control, D) maternal *PRE-Hsp70BbCas9* trans-heterozygote without serrated wing phenotype, E) maternal *PRE-Hsp70BbCas9* trans-heterozygote with serrated wing phenotype, F) paternal *PRE-Hsp70BbCas9* heterozygous control, G) paternal *PRE-Hsp70BbCas9* trans-heterozygote without serrated wing phenotype, H) paternal *PRE-Hsp70BbCas9* trans-heterozygote with serrated wing phenotype.

**Supplemental Figure S9. Sanger sequencing genotyping of *nanos* or *vasa* promoter-driven Cas9 targeting of *Serrate*.** A) Reference *Drosophila melanogaster* genome sequence of *Serrate* from NCBI Reference Sequence: NT_033777.3. gRNA target sequences are annotated in gray boxes, PAMs annotated in red boxes. Sequencing calls and corresponding chromatograms for B, F, J) *w^1118^*, C) F1 from ♀*nosCas9* x ♂*dgRNA:Ser* , D) F1 from ♀*dgRNA:Ser* x ♂*nosCas9*, E) reference (as in A) for gRNA1, F) *w^1118^*, G) F1 ♀*vasaCas9* x ♂*dgRNA:Ser*, gRNA1, H) ♀*dgRNA:Ser* x *vasaCas9*, gRNA1, J) reference (as in A) for gRNA2, K) *w^1118^*, L) ♀*vasaCas9* x ♂*dgRNA:Ser*, gRNA2, M) ♀*dgRNA:Ser* x *vasaCas9*, gRNA2.
