# Supplementary figures and images for: Polycomb response elements reduce leaky expression of Cas9 under temperature-inducible Hsp70Bb promoter in Drosophila melanogaster
Source: G3 (Bethesda). 2023 Jan 27;13(4):jkad024. doi: 10.1093/g3journal/jkad024 (PMC10085756; doi:10.1093/g3journal/jkad024)

**
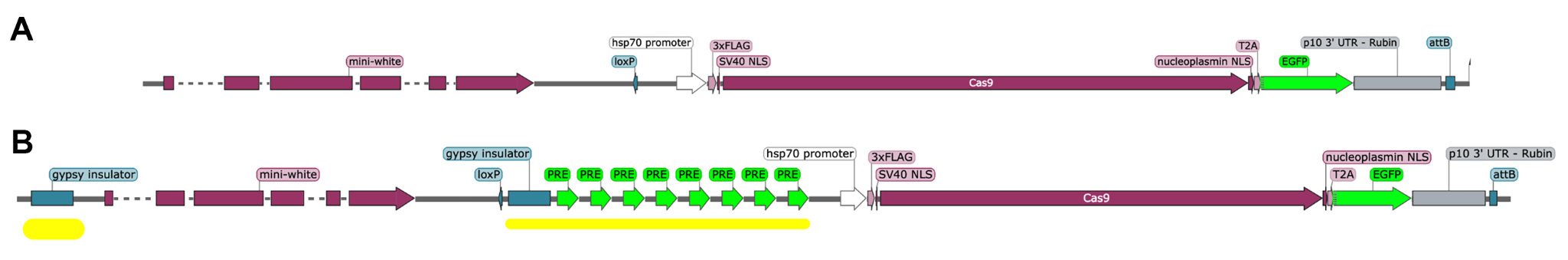
**

**Supplemental Figure S1. Transgene maps to compare *Hsp70BbCas9* and *PRE-Hsp70BbCas9.***

Supplement: jkad024_Supplementary_Data [file jkad024_supplementary_data.zip › Figure_S1_G3-2022-403941.docx]

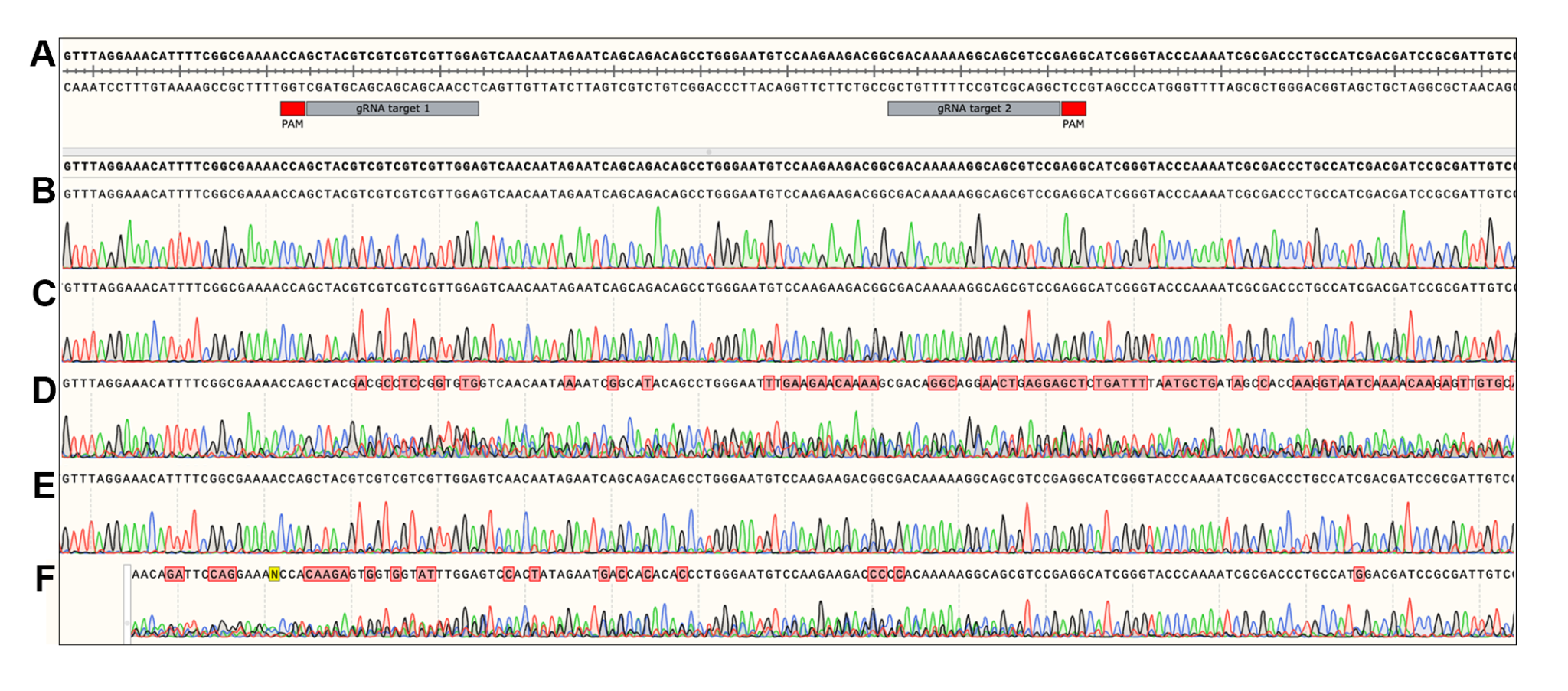


**Supplemental Figure S7. Sanger sequencing of heat-shock induced *Hsp70BbCas9* targeting of *Serrate.***

Supplement: jkad024_Supplementary_Data [file jkad024_supplementary_data.zip › Figure_S7_G3-2022-403941.docx]
